# Surgical decision-making for primary stress urinary incontinence: insights from a nationwide survey of gynecologists in Turkey

**DOI:** 10.3389/fsurg.2026.1871350

**Published:** 2026-07-16

**Authors:** İlyas Turan, Özgür Ozan Ceylan, Erhan Okuyan, Ebru Erdemoğlu

**Affiliations:** 1 Department of Gynecologic Oncology, Batman Training and Research Hospital, Batman, Türkiye; 2 Department of Obstetrics and Gynecology, Medical Park Izmir Hospital, Izmir, Türkiye; 3 Department of Obstetrics and Gynecology, Private Zilan Hospital, Batman, Türkiye; 4 Department of Obstetrics and Gynecology, Isparta City Hospital, Isparta, Türkiye

**Keywords:** laparoscopic colposuspension, midurethral sling, pelvic floor disorder, stress urinary incontinence, surgical preferences, urogynaecology

## Abstract

**Objective:**

To delineate the surgical preferences of gynecologists in the management of primary stress urinary incontinence (SUI) and to explore the extent to which clinical experience, ingrained surgical habits, and procedural volume shape these therapeutic decisions.

**Methods:**

A national, web-based cross-sectional survey was distributed among practicing gynecologists across Turkey during the 2021 calendar year. Responses from 103 clinicians were analyzed. The questionnaire captured demographic profiles, operative case volumes, frequency of diagnostic laparoscopy, intraoperative cystoscopy practices, and the preferred surgical approach for primary SUI. Respondents were stratified into two cohorts: those favoring a vaginal sling procedure (encompassing TVT, TOT, or mini-sling techniques) and those opting for laparoscopic suspension (L/S), specifically laparoscopic colposuspension. Statistical computations were performed using IBM SPSS version 20.

**Results:**

A clear majority of respondents (87.1%) gravitated toward vaginal sling techniques, while laparoscopic suspension was the preferred approach for 12.9%. Notably, surgeons favoring the L/S route demonstrated a significantly higher annual volume of open Burch colposuspension experience (*p* = 0.041). The propensity for L/S was markedly diminished among clinicians who routinely perform intraoperative cystoscopy (*p* = 0.023). The overall distribution of annual diagnostic laparoscopy volume categories differed between the two surgical groups (*p* = 0.039), with the 5–20 cases category tending to be more frequent among surgeons who favored laparoscopic suspension. While no post-hoc pairwise comparisons were performed and the finding should be regarded as descriptive, it does suggest that a moderate, sustained engagement with laparoscopic surgery. No statistically significant intergroup variations were observed regarding age, years in practice, institutional setting, or geographic region.

**Conclusion:**

The choice of surgical intervention for primary stress urinary incontinence extends beyond strict adherence to clinical guidelines; it remains intimately tied to the surgeon's individual technical repertoire and prior operative exposure. A background in advanced laparoscopy and the imprint of early surgical training emerge as potent determinants in the selection between retropubic suspension and sling-based therapies.

## Introduction

Stress urinary incontinence (SUI) ranks among the most frequently encountered clinical manifestations of female pelvic floor dysfunction and exerts a substantial detrimental impact on quality of life (1). The overarching objective of surgical intervention in this context is twofold: to alleviate or completely abolish involuntary urinary leakage while simultaneously keeping the attendant morbidity profile to an absolute minimum.

In contemporary practice, midurethral sling (MUS) procedures—whether deployed via the retropubic (TVT), transobturator (TOT), or mini-sling approach—have secured a firm foothold in the surgical armamentarium for primary SUI (2, 3). Indeed, the prevailing international guidelines unanimously endorse midurethral sling techniques as the gold standard for primary surgical correction, a status predicated on their robust objective and subjective success rates coupled with an acceptable safety margin (2, 4).

That said, the prominence of synthetic slings has not entirely eclipsed the role of mesh-free alternatives. Techniques such as laparoscopicor open Burch colposuspension continue to find application, particularly among specific patient subsets harboring reservations about mesh implantation or in settings where the surgeon's technical expertise lies elsewhere (5, 6). A growing body of literature suggests that, provided patient selection is judicious, colposuspension techniques can yield continence outcomes that rival those achieved with midurethral sling procedures (3, 6).

Moreover, the recent years of intense scrutiny surrounding mesh-related complications, the heightened emphasis on preoperative informed consent processes, and the implementation of mandatory registry and surveillance systems in several jurisdictions have collectively reshaped the landscape of surgical decision-making. It is now evident that the choice of surgical modality is sculpted not solely by the hierarchy of evidence, but equally by the prevailing regulatory climate and the nuances of surgical training infrastructure (4, 7). Within this complex milieu, survey-based field studies that capture the genuine inclinations and decision-making heuristics of practicing surgeons serve as invaluable compasses, illuminating the gap between guideline directives and everyday clinical reality (8). The present study was therefore undertaken to delineate the preference patterns of Turkish gynecologists regarding the surgical management of primary stress urinary incontinence and to elucidate the clinical and demographic factors that underpin these choices. *We hypothesized that the choice between mesh-based slings and laparoscopic suspension would be associated with quantifiable markers of surgical experience—particularly the surgeon's volume of open Burch colposuspension, annual diagnostic laparoscopy caseload, and the routine use of intraoperative cystoscopy—rather than merely with demographic or institutional factors.*

## Materials and methods

### Study design and participants

This investigation was conceived as a national, cross-sectional field survey executed during the 2021 calendar year via a dedicated online questionnaire platform. Obstetricians and gynecologists currently engaged in active clinical practice across the various geographic regions of Turkey were enrolled on an entirely voluntary basis. *Only fully certified specialists who routinely perform SUI surgery were eligible; senior residents were not included*.

### Survey instrument

The questionnaire administered to participants comprised a total of 13 discrete items. The instrument was designed to capture a comprehensive dataset encompassing: physician age, years elapsed since board certification, the nature of the employing institution (categorized as university hospital, training and research hospital, state hospital, or private sector), the specific geographic region of practice, the extent of the clinician's laparoscopic surgical experience, the routine practice of intraoperative cystoscopy, annual surgical case volume, and the preferred primary surgical approach for the management of stress urinary incontinence. The draft was reviewed for clarity and face validity by three clinicians experienced in female pelvic floor surgery and was piloted among a small number of potential respondents before final distribution. These items were selected *a priori* to test our pre-specified hypothesis that the choice between mesh-based slings and laparoscopic suspension would be associated with quantifiable markers of surgical experience—specifically the surgeon's volume of open Burch colposuspension, annual diagnostic laparoscopy caseload, and routine use of intraoperative cystoscopy—rather than with demographic or institutional factors alone.

### Data collection procedure

The distribution of the survey was orchestrated through a combination of electronic mail correspondence and individual invitation links disseminated via mobile messaging applications (WhatsApp).In order to safeguard the integrity and validity of the collected data, technical constraints were implemented to ensure that each participant could complete the survey on only a single occasion. All responses were gathered in a strictly anonymous format, and electronic informed consent was procured from every participant prior to their engagement with the questionnaire. In our case, the questionnaire was kept deliberately short and focused on concrete, factual items—such as annual case numbers, routine cystoscopy use, and a single preferred surgical approach—rather than on attitudes or psychological constructs that would require formal psychometric testing. Before distribution, the draft was reviewed for clarity and face validity by a small group of colleagues who perform incontinence surgery, and minor wording adjustments were made based on their feedback. Because invitations were distributed through multiple overlapping channels, an exact denominator could not be determined; based on the primary mailing list, the minimum response rate is estimated at approximately 5%.The study was approved by the Clinical Research Ethics Committee of Süleyman Demirel University Faculty of Medicine (Decision No: 76, Date: February 12, 2021), Isparta, Turkey. All procedures were conducted in accordance with the Declaration of Helsinki.

### Definition of study groups

For the purpose of comparative analysis, respondents were stratified into two principal cohorts predicated upon their stated primary surgical preference. Clinicians who indicated a primary reliance on retropubic (TVT), transobturator (TOT), or mini-sling techniques were amalgamated into a unified “Vaginal Sling” group. Conversely, those who favored laparoscopic Burch colposuspension were assigned to the “Laparoscopic Suspension (L/S)” cohort. To maintain conceptual homogeneity within the analytical framework, data pertaining to respondents whose primary preference involved laparotomic colposuspension, autologous fascial slings, or other ancillary procedures were excluded from the final statistical evaluation.

### Statistical analysis

All statistical computations and data processing were performed utilizing the IBM SPSS Statistics software package (Version 20). The distributional properties of continuous numerical variables were examined for adherence to normality using the Shapiro–Wilk test. In instances where the assumption of normal distribution was not satisfied, comparisons between the two independent groups were conducted employing the Mann–Whitney U test.

Associations involving categorical variables were scrutinized using the Chi-square test; in scenarios where expected cell frequencies fell below the threshold of five, Fisher's Exact test was applied as a corrective measure. For categorical variables with more than two levels, only global tests of association were performed; no adjusted pairwise post-hoc comparisons were carried out due to the limited sample size in the laparoscopic suspension group. Consequently, any observed tendencies within specific categories are presented as descriptive findings only. A two-tailed *p*-value of less than 0.05 was uniformly adopted as the benchmark for declaring statistical significance throughout the study.

## Results

A total of 103 clinicians responded to the survey invitation, of whom 101 provided complete and evaluable responses for the final analysis. The mean age of the cohort was 43.36 (9.97) years, with a mean duration of post-specialist practice spanning 16.78 (10.48) years (Table 1).

**Table 1:** Demographic and professional characteristics of the participants (*n* = 103).

Variable	Mean (SD)	Median (Min–Max)
Age(year)	43.36 (9.97)	42 (29–74)
Years in practice	16.78 (10.48)	14 (3–48)
Variable		n (%)
Institutional Affiliation		
State Hospital		13 (%12.6)
Training and Research Hospital		29 (%28.2)
University Hospital		22 (%21.4)
Private Hospital		22 (%21.4)
Private Office/Clinic		17 (%16.5)
Geographic Region		
Mediterranean		10 (%9.7)
Aegean		41 (%39.8)
Marmara		16 (%15.5)
Central Anatolia		25 (%24.3)
Black Sea		4 (%3.9)
Eastern Anatolia		4 (%3.9)
Southeastern Anatolia		3 (%2.9)
Routine Intraoperative Cystoscopy		
Yes		69 (%67.0)
No		34 (%33.0)
Laparoscopy for Gynecologic Pathology		
Yes		91 (%88.3)
No		12 (%11.7)
Primary Surgical Preference for SUI		
Vaginal sling		88 (%87.1)
Laparoscopic Suspension (L/S)		13 (%12.9)

### Overall surgical preferences

**Figure 1:**
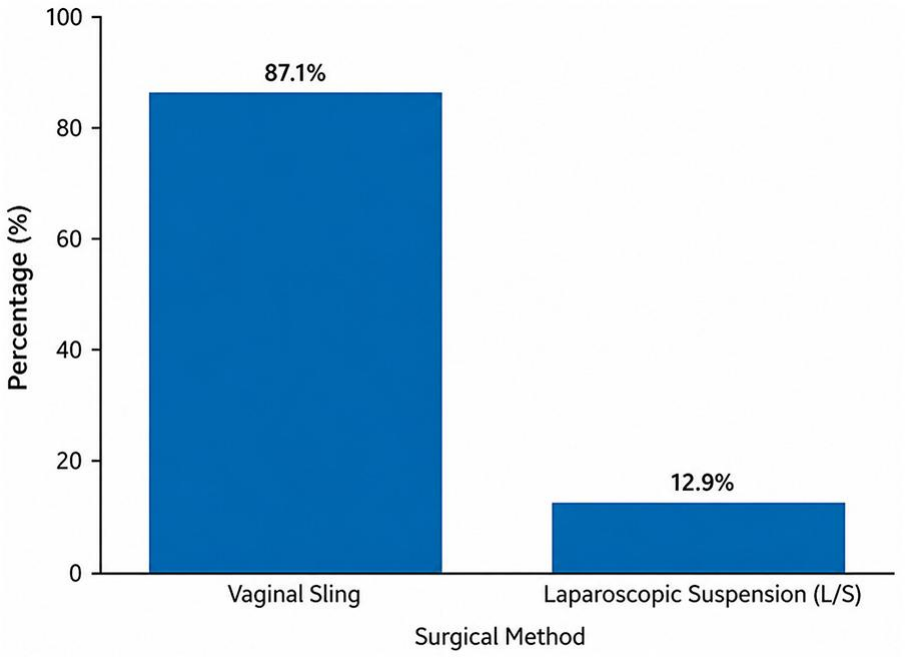
Distribution of primary surgical preference for stress urinary incontinence (*n* = 101).

**Figure 2:**
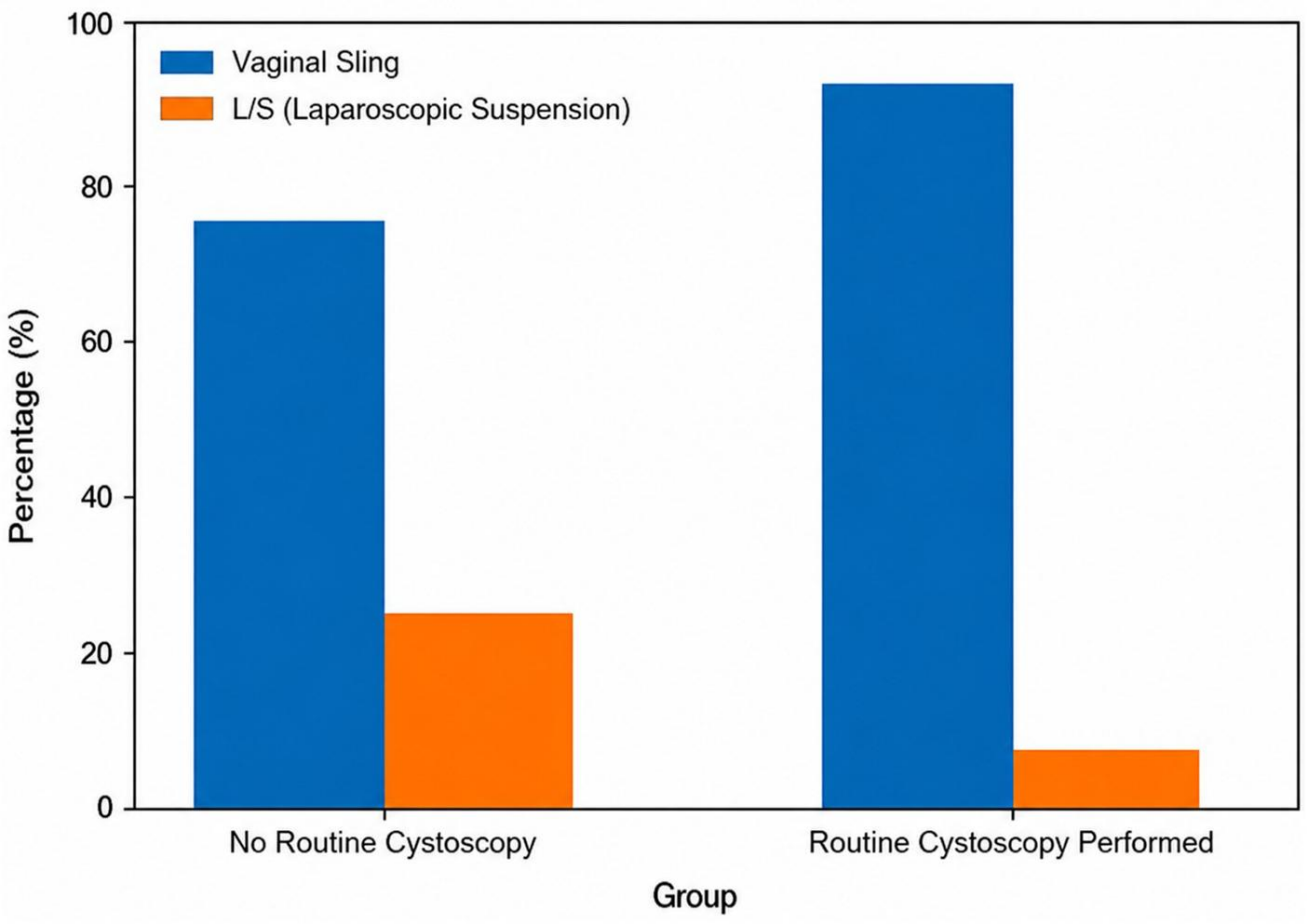
Distribution of surgical preference for primary stress urinary incontinence stratified by the practice of routine intraoperative cystoscopy (*n* = 101).

The vast majority of respondents (87.1%) identified a vaginal sling procedure as their primary surgical approach for the management of stress urinary incontinence, whereas a notably smaller fraction (12.9%) expressed a preference for laparoscopic suspension (Figures 1, 2).

### Comparative analysis of surgical cohorts

When the demographic and surgical attributes of the vaginal sling cohort were juxtaposed with those favoring laparoscopic suspension, several noteworthy divergences emerged, as detailed in Table 2.

**Table 2:** Comparison of participants preferring vaginal sling versus laparoscopic suspension (L/S) as the primary approach for SUI (*n* = 101)

Variable	VaginalSling (*n* = 88) Median (Min–Max)	L/S (*n* = 13) Median (Min–Max	*p*-value
Age (year)	43.30 (9.93)	43.69 (10.73)	0.996^a^
Yearsin Practice (year)	16.59 (10.41)	17.69 (11.25)	0.867^a^
Routine Intraoperative Cystoscopy, *n* (%)			0.023^b^
Yes	64 (%72.7)	5 (%38.5)	
No	24 (%27.3)	8 (%61.5)	
Annual Open Burch Volume	5.42 (6.21)	6.10 (2.80)	0.041^a^
Median (Min-Max)	3 (1–30)	5 (3–10)	
Annual Diagnostic Laparoscopy Volume, *n*(%)			0.039^b^
<5	36 (% 40.9)	1 (%7.7)	
5–20	35 (%39.8)	9 (%69.2)	
20–40	8 (%9.1)	2 (%15.4)	
>40	8 (%9.1)	0 (%0)	
			

Variable	Vaginal Sling (*n* = 88)	L/S (*n* = 13)	*p*-value
Institutional Affiliation, *n* (%)			0.82^b^
State Hospital	11 (%12.5)	2 (%15.4)	
Training & Research Hospital	25 (%28.4)	4 (%30.8)	
University Hospital	19 (%21.6)	3%(23.1)	
Private Hospital	20 (%22.7)	2 (%15.4)	
Private Office/Clinic	13 (%14.8)	2 (%15.4)	
Geographic Region, *n* (%)			0.74^b^
Mediterranean	9 (%10.2)	1 (%7.7)	
Aegean	36 (%40.9)	5 (%38.5)	
Marmara	13 (%14.8)	3 (%23.1)	
Central Anatolia	22 (%25.0)	3 (%23.1)	
Black Sea	3 (%3.4)	1 (%7.7)	
Eastern Anatolia	4 (%4.5)	0 (% 0)	
Southeastern Anatolia	3 (%3.4)	0 (% 0)	

a Mann–Whitney U test.

b Chi-square test or Fisher's Exact test.

A deeper inspection of the data revealed that surgeons who gravitated toward laparoscopic suspension reported a significantly higher annual volume of open Burch colposuspension procedures compared to their counterparts in the sling group (*p* = 0.041). In parallel, the propensity to adopt a laparoscopic approach was considerably dampened among clinicians who routinely perform intraoperative cystoscopy (*p* = 0.023). The overall distribution of annual diagnostic laparoscopy case-volume categories was significantly associated with surgical preference (*p* = 0.039). Specifically, the 5–20 cases category tended to be more frequent in the laparoscopic suspension group, although no formal post-hoc pairwise comparisons were perform.

The distribution of annual diagnostic laparoscopy case-volume categories differed between the two surgical groups (*p* = 0.039), though no formal post-hoc pairwise comparisons were undertaken. It is worth noting that no statistically significant intergroup differences were observed with respect to age, years in specialty practice, institutional setting, or geographic distribution.

## Discussion

The cross-sectional design of this survey, the small number of clinicians who favoured laparoscopic suspension (*n* = 13), and the possibility of unmeasured confounders all dictate that the associations we observed should be viewed as hypothesis-generating rather than definitive. This nationwide survey provides a contemporary snapshot of surgical decision-making among Turkish gynecologists for primary stress urinary incontinence (SUI), revealing an overwhelming preference for vaginal sling techniques (87.1%) over laparoscopic suspension (12.9%). This finding aligns closely with the broader international landscape, where midurethral sling (MUS) procedures have long been entrenched as the dominant surgical strategy (2, 3). The primacy of MUS in our cohort mirrors the sentiments captured in a recent large-sample global survey of urogynecologists, in which 81% of respondents designated some form of MUS—retropubic, transobturator, or mini-sling—as their preferred initial surgical approach (9). Current clinical practice guidelines, including the 2025 European Urogynaecological Association (EUGA) position statement, continue to endorse MUS as the gold standard, citing its robust efficacy and acceptable safety profile, while simultaneously emphasizing that the choice of surgical route must be tailored to individual patient risk factors and surgeon expertise (2, 4, 10).

Despite this clear consensus, our data illuminate a crucial but often underappreciated dimension of surgical practice: the imprint of individual training history and technical repertoire on therapeutic choice. The observation that surgeons favoring laparoscopic suspension (L/S) reported a significantly higher annual volume of open Burch colposuspension (*p* = 0.041) underscores a phenomenon wherein early career surgical habits appear to exert a durable, long-term influence on clinical decision-making. This is consistent with the notion that surgeons tend to gravitate toward procedures with which they possess the greatest technical fluency and confidence—a pattern that has been documented across various surgical disciplines (11, 12). In the specific context of SUI management, a recent international survey demonstrated that physicians with subspecialty training in urogynecology were significantly more likely to endorse surgical intervention and to exhibit distinct practice patterns compared with their counterparts without such fellowship experience (12).

Furthermore, the 2025 EUGA consensus explicitly acknowledges that surgeon expertise is a cornerstone of optimizing outcomes, reinforcing the critical interplay between training infrastructure and the quality of care delivered (10).

Surgeons with an intermediate annual diagnostic laparoscopy volume were more often represented in the laparoscopic suspension group, a pattern that reached statistical significance at the level of the overall distribution and should be regarded as descriptive. This finding suggests that a moderate, sustained engagement with laparoscopic surgery—even outside the specific domain of incontinence procedures—fosters a technical comfort zone that may lower the threshold for applying laparoscopic principles to anti-incontinence surgery. Conversely, the steep learning curve historically associated with laparoscopic colposuspension has been cited as a formidable barrier to its wide spread adoption (13, 14). A 2025 clinical audit from a district general hospital in the United Kingdom starkly illustrated this challenge, reporting a bladder injury rate of 17.3% for colposuspension cases—substantially exceeding the national benchmark of 2.7%—which the authors attributed to the procedural learning curve following the recent introduction of the technique at their center (14). The data from our Turkish cohort suggest that surgeons who have already navigated the early phase of the laparoscopic learning curve for other gynecologic indications may be better positioned to offer mesh-free alternatives such as LBC with greater confidence and potentially lower morbidity.

The finding that routine intraoperative cystoscopy was inversely associated with a preference for L/S (*p* = 0.023) presents an intriguing and somewhat counterintuitive observation. One might reasonably hypothesize that surgeons performing more complex retropubic dissections would be more vigilant about confirming ureteral patency and bladder integrity. Instead, our data indicate that those who employ cystoscopy as a standard adjunct are more firmly aligned with the sling group. This may reflect a broader cultural or training-based division: surgeons who favor the technically more familiar and expedient sling procedures may be more likely to adhere to rigid safety checklists, whereas those performing L/S—who by definition possess advanced laparoscopic skills—may rely on direct visualization of the retropubic space and selective, rather than routine, cystoscopic confirmation. The existing literature offers limited guidance on this specific association, highlighting an area ripe for further qualitative exploration.

The broader regulatory and medicolegal climate surrounding mesh use cannot be divorced from any contemporary discussion of SUI surgical preferences (4, 7, 15). While the FDA has maintained a more favorable stance toward MUS for SUI compared with transvaginal mesh for pelvic organ prolapse, the suspension of MUS use in countries such as Ireland (since2018) and the United Kingdom has profoundly altered the surgical landscape (14, 16). A2025 survey of Irish consultant obstetricians and gynecologists revealed that while 89.5% had performed SUI surgeries prior to the suspension—with 76.5% utilizing MUS—post- suspension, 63.2% continued to operate, pivoting primarily toward urethral bulking agents (16). Notably, over half of the Irish respondents expressed a willingness to resume MUS use should the suspension be lifted, citing perceived safety and effectiveness (16). This stands in contrast to the Turkish setting, where no such regulatory restrictions exist, and MUS remains freely available. Consequently, our findings reflect a practice environment where clinical equipoise, rather than regulatory mandate, governs the choice between sling and suspension.

It is equally important to situate our findings within the context of comparative effectiveness data. A 2024 randomized trial comparing single-incision mini-sling (SIMS) with laparoscopic Burch colposuspension (LBC) reported no significant differences in objective or subjective success rates at six-month follow-up (90% vs. 85%, *p* = 0.633, and 85% vs. 75%, *p* = 0.695, respectively), although the sling group experienced shorter operative times, catheterization duration, and hospitalization (17). These data lend credence to the assertion that, in appropriately selected patients and in the hands of experienced surgeons, mesh-free laparoscopic techniques can achieve continence outcomes that approximate those of synthetic slings (3, 5, 6, 17). Nevertheless, a 2023 updated systematic review and meta-analysis encompassing over 15,000 patients confirmed the overall superiority of MUS over Burch colposuspension in terms of both objective and subjective cure rates (odds ratio 0.51,*p* = 0.001, and OR 0.59, *p* = 0.0003, respectively) (18). The meta-analysis further highlighted the nuanced trade-offs between retropubic and transobturator approaches, with the former offering marginally higher continence rates at the expense of increased risks of bladder perforation and voiding dysfunction (18). These data underscore the message that while alternatives to MUS exist and can be effective, they should be deployed with careful patient selection and a clear-eyed appreciation of the individual surgeon's technical capabilities.

Several limitations of the present study merit acknowledgment. The survey-based design inherently relies on self-reported data, which may be subject to recall or social desirability bias. The relatively modest number of respondents who favored laparoscopic suspension (*n* = 13) may have constrained the statistical power to detect more subtle intergroup differences. Moreover, the survey was conducted in 2021, and while this remains a contemporary dataset, it may not fully capture the most recent shifts in practice patterns driven by evolving regulatory guidance and the publication of long-term mesh safety data. Nonetheless, the study's strength lies in its national scope, encompassing gynecologists from diverse institutional settings and geographic regions across Turkey. This breadth enhances the generalizability of our findings to real-world clinical practice in a middle-income country where urogynecology is largely delivered by general obstetrician-gynecologists rather than a cadre of fellowship-trained subspecialists.

In summary, the landscape of SUI surgery in Turkey is one where MUS firmly occupies the central ground, yet the surgical armamentarium is not monolithic. The choice to pursue laparoscopic suspension appears to be less a function of age, geography, or institutional affiliation, and more intimately tied to the surgeon's accumulated operative experience— particularly in open Burch procedures and intermediate-volume diagnostic laparoscopy. This pattern resonates with a growing body of evidence indicating that surgical training and subspecialty exposure are powerful determinants of practice variation (8, 12, 15). As the global conversation around mesh safety continues to evolve and as minimally invasive alternatives such as robotic-assisted colposuspension and urethral bulking gain traction, ongoing surveillance of surgeon preferences and practice patterns will be essential to ensure that patients have access to the full spectrum of safe and effective therapeutic options.

This nationwide survey of Turkish gynecologists demonstrates that vaginal sling procedures remain the unequivocal first-line surgical choice for primary stress urinary incontinence, with a clear minority favoring laparoscopic suspension. The data reveal that the decision to pursue mesh-free laparoscopic alternatives is not random, but rather is associated with specific markers of surgical experience—namely, a higher annual volume of open Burch colposuspension and a moderate annual caseload of diagnostic laparoscopy. Conversely, the routine use of intraoperative cystoscopy is more prevalent among those who adhere to the sling paradigm. These findings reinforce the principle that surgical decision-making in SUI extends well beyond the dictates of clinical guidelines; it is a deeply personal calculus shaped by the surgeon's training heritage, technical fluency, and the subtle but enduring influence of early career surgical habits. Surgeons with an intermediate annual diagnostic laparoscopy volume were more often represented in the laparoscopic suspension group, a pattern that reached statistical significance at the level of the overall distribution and should be regarded as descriptive.

### Limitation

The modest estimated response rate also raises the possibility of non-response bias, and the preferences of non-responders may differ from those captured here. The small comparator group precluded multivariable modelling, so the reported associations are unadjusted and should be regarded as exploratory.

## Conclusion

*These patterns suggest that further prospective research into the interplay between surgical training, ongoing case-volume exposure, and procedure selection is warranted to optimize both patient counselling and surgical outcomes.*

## Data Availability

The raw data supporting the conclusions of this article will be made available by the authors, without undue reservation.
